# Editorial: Current proceedings in magnetocardiology—past, present, future

**DOI:** 10.3389/fcvm.2024.1444963

**Published:** 2024-08-23

**Authors:** J-W. Park, D. Dischl, K. Aschbacher, D. Kranz, J. C. Rieß, S-W. Kim, J. Brachmann, S. Treskatsch, B. Heidecker, U. Landmesser, N. Wessel

**Affiliations:** ^1^ German Heart Center Berlin, Department of Cardiology, Angiology and Intensive Care Medicine, Charité – Universitätsmedizin Berlin, Berlin, Germany; ^2^ SandboxAQ, Palo Alto, CA, United States; ^3^ Department of Physics, Humboldt Universität zu Berlin, Berlin, Germany; ^4^ Chung-ang University Gwangmyeong Hospital, Heart & Brain Hospital, Gwangmyeong-si, Gyeonggi-do, Republic of Korea; ^5^ Medical School REGIOMED, Coburg, Germany; ^6^ Department of Anesthesiology and Intensive Care Medicine, Charité-Universitätsmedizin Berlin, Corporate Member of Freie Universität Berlin and Humboldt Universität zu Berlin, Berlin, Germany; ^7^ Department of Human Medicine, MSB Medical School Berlin GmbH, Berlin, Germany

**Keywords:** magnetocardiogram (MCG), cardiac disease diagnosis, monitoring, myocarditis, magnetoionography

**Editorial on the Research Topic**
Current proceedings in magnetocardiology—past, present, future

This special issue on “Current Proceedings in Magnetocardiology—past, present, future” explores the latest advancements and applications of magnetocardiography (MCG) in clinical settings. The included papers cover a wide range of topics, from the use of MCG in diagnosing and monitoring cardiac conditions like myocarditis and cardiomyopathies to the technological advancements enabling unshielded MCG use in everyday clinical practice. Each study contributes to our understanding of MCG's diagnostic capabilities, offering insights into its non-invasive nature, efficiency, and potential for enhancing cardiac care. The editorial describes the newly discovered magnetoionography (MIG), which for the first time enables us to non-invasively measure the membranous ion currents of action potential as well as the subcellular Ca2 + transient. Finally, the editorial is rounded off with the vision of the future that we can and must expect when artificial intelligence (AI) is applied to MCG/MIG.

Golpour et al. discuss the use of MCG to monitor therapy in a patient with cardiac transthyretin amyloidosis (ATTR), treated with tafamidis. After treatment, the patient, an 83-year-old woman, showed improvement not only in quality of life, but also in MCG measures, thereby suggesting MCG's potential as a monitoring tool. Initial MCG measurements of the patient indicated a pathological state, which normalized four months into therapy and showed improvement over the treatment course. Despite slight increases in the MCG vector (1) after 27 months, overall trends indicated a beneficial response to therapy, paralleled by improvements in exercise tolerance and quality of life. However, there were no significant changes in cardiac wall thickness or echocardiographic parameters despite the observed MCG improvements. The case prompts a discussion on the integration of MCG with conventional diagnostic methods and the potential for MCG to enhance the management of cardiac amyloidosis and other non-ischemic cardiomyopathies.

Suwalski et al. present a case of a 53-year-old male with recurrent inflammatory cardiomyopathy, diagnosed and monitored by MCG. Despite standard diagnostic approaches—such as endomyocardial biopsy, cardiac magnetic resonance, and positron emission tomography-computed tomography, providing valuable insights—their limitations necessitate alternative methods. MCG, in this context, emerges as a complementary tool, offering the benefits of non-invasiveness, lack of radiation exposure, and real-time monitoring capabilities. The patient's journey, marked by two episodes of inflammatory cardiomyopathy over a two-year span, showcases MCG's efficacy in diagnosing and guiding immunosuppressive treatment. The integration of MCG into the standard care protocol for inflammatory cardiomyopathy could enhance patient outcomes through more precise and timely interventions.

Musigk et al. provide a detailed overview of the inflammatory spectrum of cardiomyopathies, covering various etiologies such as viral, bacterial, and immune-mediated myocarditis. It emphasizes the complex interplay of different cell types, cytokines, and chemokines in the development and progression of these conditions. The review highlights the importance of understanding the intricate immune mechanisms involved in developing more effective, targeted therapies. It also discusses the gaps in current knowledge and the need for new translational approaches to improve early detection methods and treatments for inflammatory cardiomyopathies. The comprehensive analysis presented in this review underscores the varied nature of these diseases and the crucial role of the immune system in their pathogenesis.

Pille et al. assessed the efficacy of MCG in distinguishing patients with suspected myocarditis from healthy controls using Kullback–Leibler entropy (KLE) for analyzing cardiac magnetic field map topologies. KLE is a measure from information theory that quantifies the difference between two probability distributions, and which can be applied in 2-dimensions to quantify the topological differences between two magnetic field maps. Results demonstrated that MCG, particularly through the STT segment analysis, can reliably differentiate between the two groups, achieving good sensitivity and specificity. This suggests MCG's potential as a non-invasive diagnostic tool for myocarditis, complementing traditional methods and improving early detection and treatment strategies.

Brisinda et al. review the evolution and current status of unshielded MCG, detailing the technological advancements that have allowed for more practical and less expensive MCG systems. These developments enable the use of MCG in unshielded, everyday clinical settings, overcoming previous limitations that required expensive, shielded environments. The authors discuss various sensor technologies such as optically pumped magnetometer MCG (OPM-MCG), and the potential for future applications in clinical diagnostics and monitoring, emphasizing the importance of MCG in non-invasive cardiac assessment. However, prior to the transfer of SQUID MCG findings on OPM-MCG, an equivalence test in form of head-to-head comparison, is mandatory.

Her et al. (2) showcase MCGs efficacy in detecting ischemic changes in patients with coronary artery disease, highlighting its superior sensitivity compared to traditional methods like ECG, especially under conditions of stress. MCG demonstrated high diagnostic accuracy, with various studies showing its potential to differentiate patients with stable coronary artery disease and those with acute coronary syndromes from healthy individuals, even in unshielded environments. The results reinforce MCG's potential as a valuable non-invasive tool in the diagnosis and monitoring of ischemic heart disease.

Wessel et al. investigated the potential of MCG at rest for predicting mortality in patients with acute chest pain (ACP). Key findings include that heart rate-corrected QT (QTc) prolongation, decreased repolarization reserve, and increased serum creatinine are significant predictors of mid-to-long-term mortality in ACP patients. These parameters showed high sensitivity (90.9%) and specificity (85.6%) for predicting cardiac death, highlighting MCG's value as a non-invasive tool for risk stratification and supporting the development of new prevention strategies.

The contribution of Wessel et al. is using MIG in their analysis. MIG emerges as a promising new technique extending the capabilities of traditional MCG by focusing on the ion-specific currents within the heart, potentially offering deeper insights into cardiac electrophysiology. This advanced technique potentially provides detailed information on cardiac ion channel functions and their disturbances, pivotal in diagnosing and understanding arrhythmias and other cardiac abnormalities. By measuring the magnetic fields generated by these ion currents, MIG could present a groundbreaking approach in cardiac diagnostics, holding promise for improved precision in identifying and monitoring cardiac diseases.

Most clinically relevant sarcolemmal ion currents can be characterized and quantified by MIG due to their specific polarity, speed, rotational behavior, and distinct occurrence intervals during the electromagnetic heart cycle. For example, the depolarizing Na + current, which flows during the QRS interval, is magnetically dipolar, fast (2–3 m/s) (3), and exhibits strong rotational movement because it travels via the fast-track Purkinje fibers. Conversely, the repolarizing transient outflow current (Ito), responsible for early repolarization, appears monopolar in frontal projections as its transmural direction of flow is perpendicular to the projection. Additionally, this magnetic vector does not rotate and occurs within the time interval of phase 1 of the action potential (AP). Beyond measuring sarcolemmal currents such as the repolarization currents IKr and IKs, and the depolarization current ICaL, MIG can also measure subcellular Ca2 + transients. This is due to MIG's ability to detect electromagnetic signals from the heart in a contactless manner. Therefore, the currents measured by MIG during phases 2 and 3 of the action potential include both sarcolemmal currents and subcellular Ca2 + transients. These Ca2 + transients involve systolic Ca2 + release from the sarcoplasmic reticulum through Ryanodine Receptor type 2 (RyR2) into the cytosol, and diastolic Ca2 + uptake from the cytosol by the sarco-endoplasmic reticulum Ca2+ ATPase (SERCA) pump into the sarcoplasmic reticulum. Both currents are dipolar because the directions of Ca2 + movement during systole and diastole are parallel to the shortening of heart muscle fibers.

In contrast, the electrocardiogram (ECG), which picks up electrical signals transmitted through biological tissues, cannot detect subcellular Ca2 + transients. This is because these currents flow solely within the cardiomyocyte, and therefore, the electromagnetic signals from these currents are isolated by the cell membrane.

Evolution has adopted positively charged Ca2 + and negatively charged phosphate ions as the two primary signaling elements of cells. Ca2 + signaling influences every aspect of a cell's life and death. Being the most tightly regulated ion within all membrane-bound organisms, Ca2 + binds to thousands of proteins, thereby affecting changes in localization, association, and function (4). This underscores the versatility of Ca2 + as a signaling molecule. The heart muscle-wide changes in intracellular Ca2 + concentration are initiated and coordinated by the electrical depolarization of the cardiomyocyte sarcolemma through the action potential. Ca2 + signaling pathways also play a role in development and pathological conditions. The prevalence of altered Ca2 + homeostasis in cardiovascular diseases underscores the central importance of Ca2 + in heart function (5, 6).

Several factors can disrupt calcium signaling, potentially impairing Ca2 + handling. These include oxidative stress (7), ischemia and hypoxia (8), heart failure and cardiomyopathies (9), inflammation (10), metabolic disorders (11), and exposure to drugs and toxins (12). Genetic factors also play a role (13). Any abnormalities in these areas can serve as early indicators of myocardial dysfunction, irrespective of the specific underlying pathophysiological cause.

**Figure 1:**
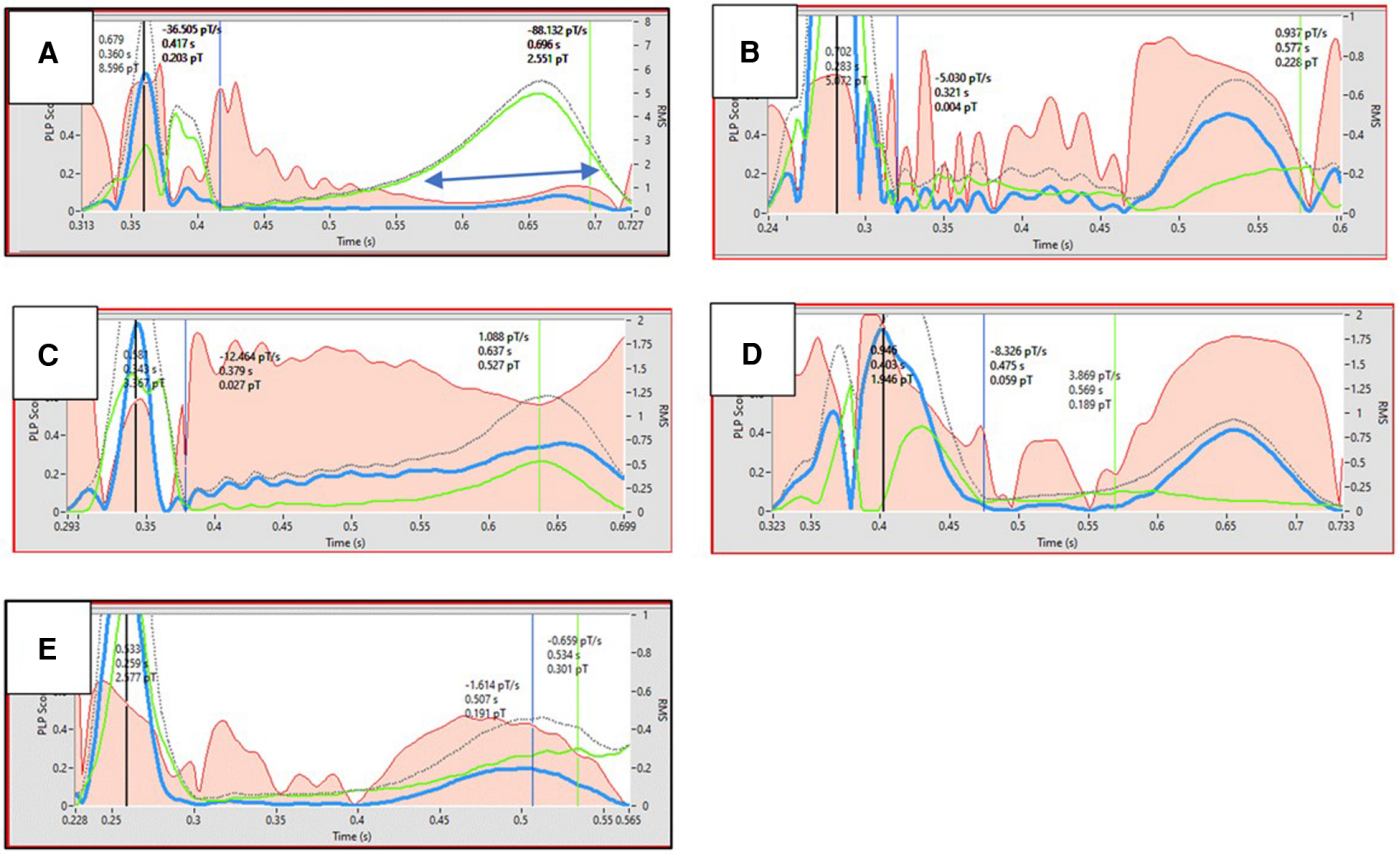
Black dotted line represents the action potential. Red area represents the monopolarity index (instantaneous percentage of monopolar currents). Ordinate shows the scaling of magnetic field strength. Double blue arrows indicates the time interval where Ca2 + transient flows. The figure illustrates examples of Ca2 + transient dysfunction in various diseases **(B–E)** and in a healthy state **(A)** Ca2 + transients are shown in green. Dysfunctional Ca2 + transient is characterized by Ca2 + transient max suppression **(B–E)**, desynchronity between Ca2 + transient max and action potential Tmax **(B,D,E)**, and reduced Ca2 + uptake velocity **(B,D,E)**. A. Healthy (26yrs, male, marathon runner); B. Old age (76yrs, male, healthy); C. Insulin dependent Diabetes mellitus (76yrs, male); D. HFrEF (46yrs, male); E. Post COVID disease (56yrs, female).

Building on the foundation of MCG, MIG introduces a novel dimension to cardiac diagnostics by enabling the non-invasive tracking of specific ionic movements, particularly calcium and potassium ions, which are crucial in cardiac rhythm management (Figure 1). The technology's potential to map these ion currents accurately paves the way for a new era in personalized cardiac care, facilitating early detection of diseases and the assessment of treatment efficacy. As MIG develops, it may offer a unique window into the heart's ionic processes, leading to better clinical outcomes through tailored therapeutic interventions.

Although the promising future of MIG/MCG, namely quantum sensing at room temperature, contactless, affordable, portable, and even wearable devices for unshielded MCG, suitable for mass screening, seems within reach, equivalence testing with He-cooled SQUID sensors in a shielded chamber, which remains the gold standard in terms of signal-to-noise ratio, in the form of a head-to-head comparison is still pending.

A significant advantage of MCG is its capacity to encode a wealth of information across numerous channels, or high signal entropy. At the same time, this entropy also presents a challenge, as traditional analysis methods fail to fully exploit it. For instance, a prevalent approach in MCG analysis involves averaging signals across channels to produce a composite representation. This composite is often used, in part to facilitate denoising via signal processing methods. However, it also obscures potentially useful patterns such as electrical alternans or autoregressive variability (14–16). An alternative approach could leverage artificial intelligence (AI) and deep learning algorithms to denoise the signal without requiring a composite, thereby enabling the extraction of additional valuable diagnostic information. However, particularly in unshielded environments, where the challenges of denoising are substantial, further research is needed to compare traditional signal processing methods with deep learning approaches.

Another likely advantage of AI applied to MCG is the capability to capture complex morphologic patterns over time within each channel as well as the spatial information across channels. The existing literature (e.g., Pille et al.) highlights the value of tracking the changes in the topologic map over time. However, this method likely inflates the sensitivity and specificity, due to multiple comparisons of the KL test over each temporal snapshot. Moreover, it fails to model the intrinsic relationships between subsequent “snapshots” of the magnetic field map in the temporal sequence or capture much specificity with respect to spatial patterning. In contrast, deep learning architectures such as convolutional and recurrent neural networks excel at capturing spatial-temporal patterns, while utilizing a train-test split that also mitigates issues of multiple comparisons. Arguably, one of the most significant challenges in the application of AI to MCG for diagnosis is simply the dearth of large datasets.

The ultimate vision for the application of AI to MCG is the ability to provide physicians with a real-time clinical decision aid and novel insights. While deep learning excels in predictions from complex sensor or image data, the “Achilles heel” of such approaches may be that the results can be difficult to explain to clinicians and to map onto known pathophysiology, to guide treatment. Finally, deploying AI models in real-world settings will require frameworks to tackle the many sources of heterogeneity that challenge model generalization, and thereby address issues of AI equity, transparency, and trustworthiness.
